# Photocatalytic hydrogen production and storage in carbon nanotubes: a first-principles study†

**DOI:** 10.1039/d2ra02349k

**Published:** 2022-06-08

**Authors:** Xiaohan Song, Hongxia Bu, Yingcai Fan, Junru Wang, Mingwen Zhao

**Affiliations:** Shandong Institute of Advanced Technology Jinan Shandong 250100 China; College of Physics and Electronic Engineering, Qilu Normal University Jinan Shandong 250200 China; School of Information and Electronic Engineering, Shandong Technology and Business University Yantai Shandong 264005 China; Department of Physics, Yantai University Yantai Shandong 264005 China; School of Physics and State Key Laboratory of Crystal Materials, Shandong University Jinan Shandong 250100 China zmw@sdu.edu.cn

## Abstract

As it is a promising clean energy source, the production and storage of hydrogen are crucial techniques. Here, based on first-principles calculations, we proposed an integral strategy for the production and storage of hydrogen in carbon nanotubes *via* photocatalytic processes. We considered a core–shell structure formed by placing a carbon nitride nanowire inside a carbon nanotube to achieve this goal. Photo-generated holes on the carbon nanotube surface promote water splitting. Driven by intrinsic electrostatic field in the core–shell structures, protons produced by water splitting penetrate the carbon nanotube and react with photo-generated electrons on the carbon nitride nanowire to produce hydrogen molecules in the carbon nanotube. Because carbon nanotubes have high hydrogen storage capacity, this core–shell structure can serve as a candidate system for photocatalytic water splitting and safe hydrogen storage.

## Abstract 2

The production and storage of hydrogen in CNNW/CNT core–shell structures *via* photocatalytic processes.

## Graphical abstract

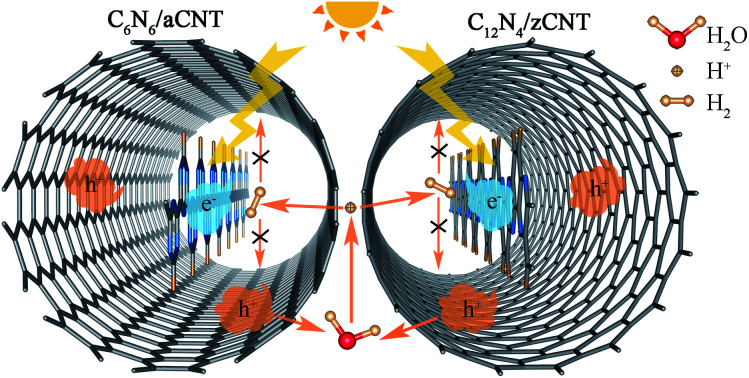

## Introduction

Hydrogen energy has attracted a great deal of attention as a sort of clean energy resource. The hydrogen production *via* photocatalytic overall water splitting is a regenerative, eco-friendly, and inexhaustible approach, where photocatalysts play a key role.^1–3^ The photocatalysts employed in these processes should have high absorption ability to light, suitable band gap and band edges that match with the redox potentials of water splitting, and high redox ability of the photogenerated carriers. So far, a large number of photocatalysts, such as metal-free photocatalysts, metal oxides, metal chalcogenides and so on, have been developed for hydrogen production from water splitting.^4–12^ Among these semiconductor catalysts, carbon-based materials were intensively studied because of their excellent catalytic performance.^13–20^ Graphitic carbon nitride (g-C_3_N_4_) with proper band edges has been widely studied, but its photocatalytic performance is hindered by poor light-harvesting ability and low charge mobilities.^21–28^ Fortunately, the construction of heterojunctions or modifications can improve the aforesaid problems.^29^

The separation and storage of the generated hydrogen molecules in the photocatalytic water splitting processes are also crucial. In order to avoid the reverse reaction and possible dangerous explosion, hydrogen should be isolated from the oxygen products during water oxidation. Recently, Yang *et al.* reported that photocatalytic hydrogen generation and hydrogen storage can be realized in a two-dimensional multilayer system, where carbon nitride monolayer is sandwiched between two monolayers of graphene.^30^ The key is that the outer graphene layers allow only protons to pass through, separating the production and storage of hydrogen and oxygen.^31^ But the storage rate of hydrogen is limited by the interlayer distance, as effective interlayer charge transfer distance between 2D material is ∼7 Å.^32,33^

Carbon nanotubes (CNTs) have been widely studied for hydrogen storage due to abundant carbon elements, light mass density, highly and controllable porous structure, and good interaction between carbon and hydrogen molecules.^34^ But one challenge in storing hydrogen inside CNTs is how to put hydrogen in it. Notably, various nanowires have been successfully encapsulated in carbon nanotubes, such as carbon chain, metal nanowires, nitride nanowires, carbide nanowires and so on.^35–39^ Supposing a photosensitive material is placed inside a CNT, it will conduct photocatalytic reaction and generate hydrogen molecules inside the CNT. Because only the protons are allowed to pass through the wall of CNT,^31^ the produced hydrogen molecules are separated from oxygen, achieving safe and stable storage of hydrogen inside CNTs. The double-walled nanotubes can also achieve this goal.^40^

Using first-principles calculations, we proved the rationality and feasibility of this strategy in CNT containing a carbon nitride nanowire (CNNW). The electronic structures, separation of the photo-generated carriers and the subsequent chemical reactions in this photocatalytic system were systematically investigated. Our calculations showed that the oxygen evolution reaction (OER) and hydrogen evolution reaction (HER) take place on the outside wall of CNTs and the inner CNNWs, respectively, driven by the photo-generated carriers, enabling the separation and storage of hydrogen in CNTs. In view of the high hydrogen storage capacity of carbon nanotubes, this core–shell structure can serve as a candidate system for photocatalytic water splitting and safe hydrogen storage.

## Computational methods

Our density functional theory (DFT) calculations were performed by using the Vienna Ab-initio Simulation Package (VASP) code.^41^ The electron–ion interactions and the electron–electron exchange–correlation were respectively described by the projector augmented wave (PAW) method^42^ and the generalized gradient approximation (GGA)^43^ in the form of Perdew–Burke–Ernzerhof (PBE) functional.^44^ The hybrid Heyd–Scuseria–Ernzerhof (HSE06) functional^45^ was used for accurately calculating the band structures. The van der Waals (vdW) interaction between CNT and CNNW was described by the DFT-D2 Grimme's method.^46^ The energy cutoff was set to 520 eV. The conjugate gradient (CG) algorithm was used to fully relax all the atom positions and the lattice constants. The convergence criteria for energy and force was 10^−5^ eV and 0.01 eV Å^−1^, respectively. A periodic boundary condition was applied along the axial (*z*-) direction of the CNT and CNNW, while a vacuum space of about 30 Å was included along the lateral (*x*- and *y*-) direction to avoid the interaction between the adjacent images. The Brillouin zone was sampled with the Monkhorst-Pack *k*-points meshes of 1 × 1 × 15 for CNTs, 1 × 1 × 7 for CNNWs and the core/shell structures, respectively.^47^ The electron transfer between the inner CNNWs and the outer CNTs was calculated by means of Bader analysis.^48^ We use the climbing image nudged elastic band (CNEB) method,^49^ which was implemented in the VASP transition state tools, to determine the energetic minimal path profiles for the water splitting at the CNTs surface. The nonadiabatic molecular dynamics (NAMD) simulations were also performed to examine the spatial evolution of photogenerated carriers based on the Hefei-NAMD code.^50^ The detailed description of NAMD can be found in the ESI.†

The HER pathways were calculated according to the electrochemical framework developed by Nørskov *et al.*,^51^ the HER overall two-electron transfer reaction process in an acid electrolyte can be written as:H^+^ + e^−^ + * ⇌ H* ⇌ 1/2H_2_(g) + *where * denotes an adsorption site on the CNNWs, H* are adsorbed intermediate, and (g) refer to gas phases. The free energies of the steps were calculated by using the following equationΔ*G* = Δ*E* + Δ*E*_ZPE_ − *T*Δ*S* + Δ*G*_U_where the Δ*E* is the DFT computed adsorption energy, the Δ*E*_ZPE_ is the zero-point energy difference and Δ*S* is the entropy difference between the initial state and the final state of the reactions. Δ*G*_U_ = −e*U*; in which *U* is the electrode applied potential. Table S1† shows the calculated *E*_ZPE_ values in this work. The theoretical overpotential (*η*) for HER can be determined from the free energy change (Δ*G*_H*_) of the HER processes:*η* = −|Δ*G*_H*_|/e

## Results and discussion

**Fig. 1:**
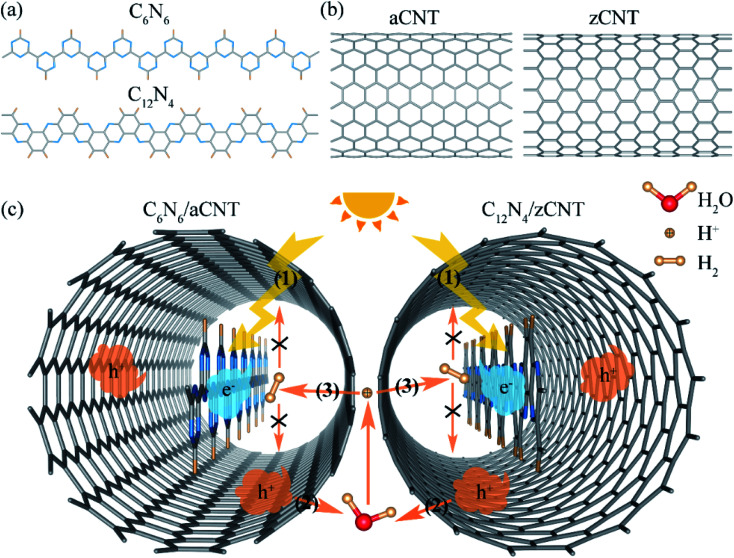
(a) The models of carbon nitride nanowires: C_6_N_6_ and C_12_N_4_. (b) The models of carbon nanotubes: (10,10) armchair CNT (aCNT) and (17,0) zigzag CNT (zCNT). (c) The solar-driven photocatalytic water splitting and safe hydrogen storage scheme: (1) separation of photo-generated carries; (2) photo-generated carriers promote water splitting to generate proton (H^+^); (3) proton penetrate the wall of CNTs, hydrogen generation and storage. Blue, grey, orange and red beads represent N, C, H (H^+^) and O atoms, the blue and orange clouds are photo-generated carries (e^−^ and h^+^), and the orange arrows denote the particles migration.

To achieve photocatalytic water splitting, the band gap of the photocatalysts should be at least 1.23 eV, which correspond the energy difference between hydrogen reduction potential (H^+^/H_2_) and water oxidation potential (O_2_/H_2_O). In this work, we took two CNNWs, C_6_N_6_ and C_12_N_4_ (Fig. 1a) as the photocatalysts. The lattice constants of the C_6_N_6_ and C_12_N_4_ nanowires are respectively 7.13 Å and 8.32 Å. The bandgaps, 3.65 eV (for C_6_N_6_) and 2.51 eV (for C_12_N_4_), and the band edge alignment as shown in Fig. S1 and S2,† fulfill the requirement of photocatalytic water splitting. The optical adsorption peaks of these CNNWs reside at the ultraviolet light regime, as shown in Fig. S3.†

We considered two types of CNTs, (10,10) armchair CNT (aCNT) and (17,0) zigzag CNT (zCNT), as shown in Fig. 1b. The diameters of aCNT and zCNT are respectively 13.64 Å and 13.42 Å. In order to effectively reduce lattice mismatch, the core–shell structures were constructed by using similar shaped materials along radial direction. C_6_N_6_ and C_12_N_4_ nanowires were placed in the two CNTs, respectively, which are denoted as C_6_N_6_/aCNT and C_12_N_4_/zCNT, as shown Fig. 1c. The lattice parameters of C_6_N_6_/aCNT and C_12_N_4_/zCNT were set to be *c* = 7.27 Å and 8.44 Å, corresponding to the average values of the CNNWs and CNTs. We define the formation energy as *E*_f_ = *E*_CNNW/CNT_ − *E*_CNNW_ − *E*_CNT_, where *E*_CNNW/CNT_, *E*_CNNW_ and *E*_CNT_ represent energies of the CNNW/CNT structures, CNNW and CNT, respectively. Our calculations showed that C_6_N_6_/aCNT and C_12_N_4_/zCNT have similar minimum equilibrium interfacial distances between CNT and CNNW of 3.60 Å and 3.37 Å and formation energies of 0.89 eV and 0.14 eV, suggesting the weak vdW interaction between the two components. The semiconducting features of the CNNWs are well preserved with reduced bandgap in the core–shell structures (Fig. S1†). And these core–shell structures have direct bandgaps at the *Γ* point, the conduction band minimum (CBM) and valence band maximum (VBM) is dominated by CNNWs and CNTs, respectively. The range of light absorption has also been extended to visible and ultraviolet light (Fig. S3†).

The production and storage of hydrogen in these core–shell structures *via* photocatalytic processes are illustrated in Fig. 1c. (1) The core/shell structure generates excitons by absorbing visible or ultraviolet light. The subsequent charge separation *i.e.*, electrons reside on the inner CNNWs while holes transfer to outer CNTs, facilitates the following reactions. (2) The holes on CNTs (CNTs_Cv_) facilitate water adsorption to defect sites on CNTs surface and subsequent water splitting to generate protons (H^+^). (3) Under the action of electrostatic attraction driving force, the generated H^+^ penetrate the wall of CNTs and reach the N sites of CNNWs. (4) Driven by the photo-generated electrons in CNNW, HER takes place, producing H_2_ molecules inside the CNT. The produced H_2_ molecules cannot penetrate the wall of CNTs and thus are retained inside CNTs, realizing the purpose of safe capsule storage.

We first investigated the separation of photo-generated carriers in the photocatalytic systems. The direction of charge transfer can be predicted from the work function (WF) of the materials. Electrons prefer to transfer from a low WF material to a high WF material.^52^ Our calculations showed that CNNWs have higher WFs than those of CNTs, as shown in Fig. S4.† This is also consistent with Bader analysis for the neutral core–shell structures at the equilibrium state. The inner C_6_N_6_ and C_12_N_4_ NWs gain 0.082 and 0.068 electrons from the CNTs, respectively, exhibiting that the electrons are well separated from the hole carriers, as shown in Fig. S5.† The electron transfer also induces a built-in electric field in the core–shell structures, which promotes the subsequent separation of the photo-generated carriers and then proton penetration through the wall of CNTs.

**Fig. 2:**
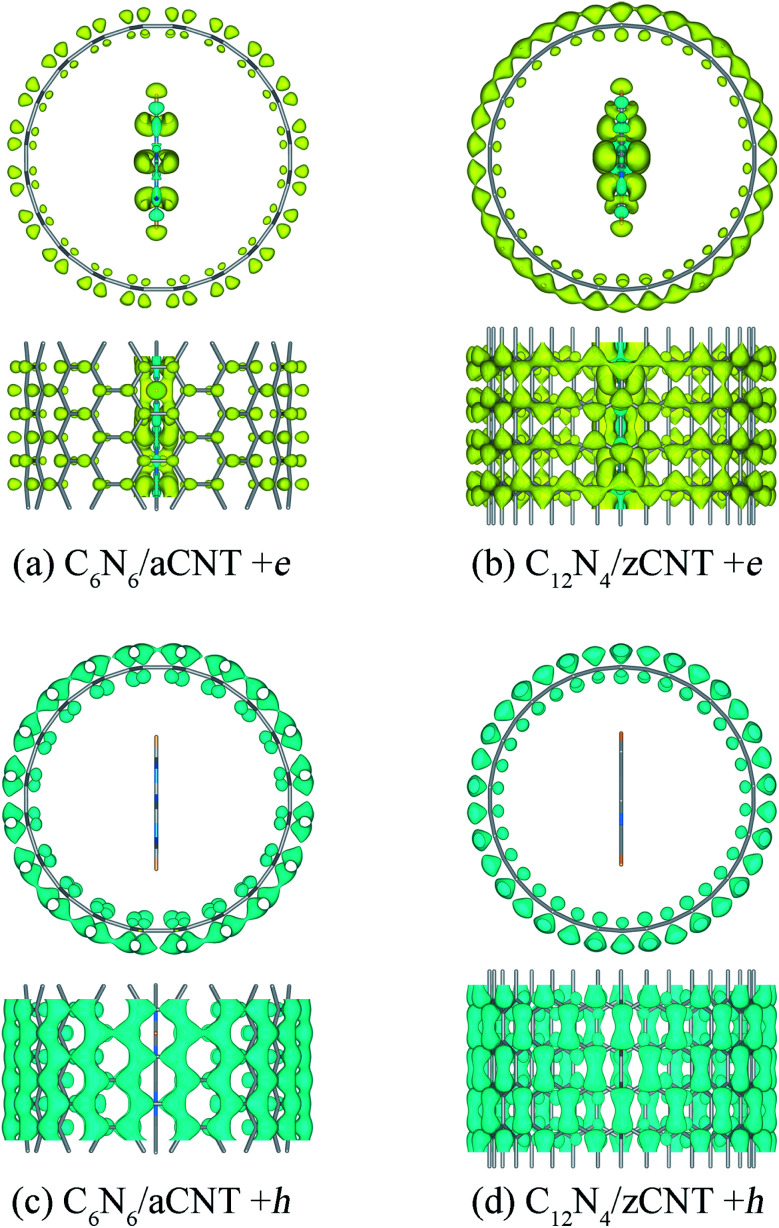
Charge density difference of the C_6_N_6_/aCNT and C_12_N_4_/zCNT structures with one extra electron (a and b) and hole (c and d). The isovalue was 2 × 10^−3^*e*/Å^3^. Yellow regions and blue regions indicate electron accumulation and loss, respectively.

Additionally, we also mimicked the spatial distribution of the photo-generated electrons in the CNNW/CNT structures. We plotted the band edge alignment of the CNNW/CNT structures (Fig. S2b and c†). The results showed CNNW/CNT structures have the staggered band alignment, in which electron and hole carriers are spatially separated. Band-decomposed charge density distributions for the CBM and VBM of CNNW/CNT structures also showed that the VBM are localized at the CNTs and the CBM are contributed by the CNNWs (Fig. S6†). The inner CNNWs accumulate electrons, whereas outer CNTs always collect hole carriers, as shown in Fig. 2. Bader charge analysis indicates that about 0.246 and 0.275 e^−^ accumulate on the C_6_N_6_ and C_12_N_4_ NWs, as an additional electron was added into the unit cell of the photocatalytic systems, while the CNTs could collect 1.031 and 1.072 h^+^ for the hole-doped systems, respectively. The NAMD simulations showed that the hole on CNTs would almost not transfer to the CNNWs within 0.5 ps, while more than 50% electron carriers on the CNTs would transfer to the CNNWs within 0.3 ps and 0.4 ps respectively for C_6_N_6_/aCNT and C_12_N_4_/zCNT (Fig. S7†). This suggested that photo-generated electrons and holes are distributed on CNNWs and CNTs respectively.

**Fig. 3:**
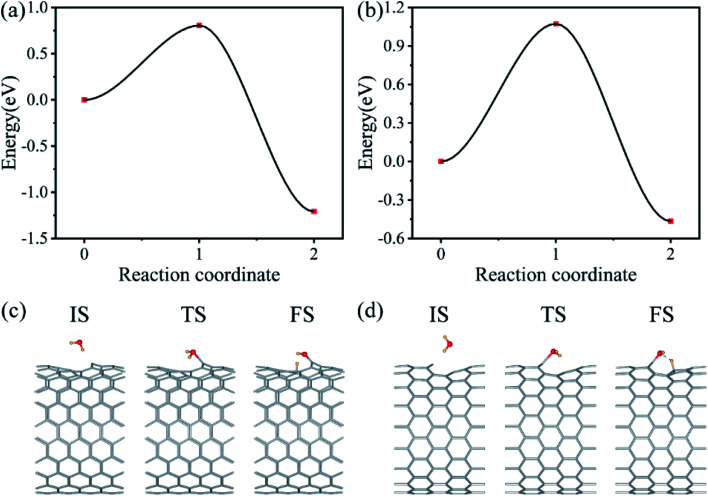
The energy profiles for water splitting at the (a) aCNT_Cv_ and (b) zCNT_Cv_ surface. The corresponding structural configurations of the reaction path for water molecule catalyzed by (c) aCNT_Cv_ and (d) zCNT_Cv_.

Water splitting processes starts with the water molecule adsorption on the surface of CNTs. We only need to produce protons in this step. The introduction of vacancy defects and single-atom catalysts, such as Fe, Co, Ni, Cu, Zn *etc.*, on the wall of CNTs can improve the activity of the photocatalytic systems and lifetime of photogenerated carriers.^53^ Here we introduced a vacancy defect on the wall of CNTs (CNTs_Cv_) as an example. The adsorption energy of H_2_O molecule on the vacancy defect of CNTs was defined as *E*_ad_ = *E*_CNT_ + *E*_H_2_O_ − *E*_H_2_O@CNT_, where *E*_CNT_, *E*_H_2_O_ and *E*_H_2_O@CNT_ represent the energies of a CNT, H_2_O and the complex of CNT and H_2_O, respectively. Our calculations showed that a water molecule can be stably adsorbed on the vacancy defect of the CNTs with the adsorption energies of 1.46 eV and 0.74 eV for the aCNTs_Cv_ and zCNTs_Cv_, respectively. Furthermore, the water splitting energy barrier (*E*_b_) can be significantly reduced by vacancy defects. The transition state calculations based on the CNEB method revealed that the *E*_b_ values of the H_2_O adsorbed on the defective CNTs are only 0.81 eV (aCNTs_Cv_) and 1.07 eV (zCNTs_Cv_), as shown in Fig. 3. Such low energy barriers facilitate the subsequent photocatalytic water splitting processes.

**Fig. 4:**
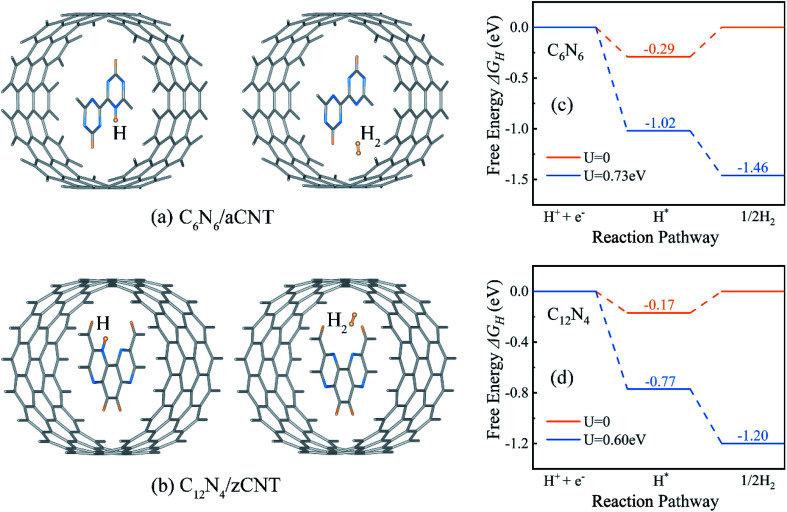
Optimized configurations of (a) C_6_N_6_/aCNT and (b) C_12_N_4_/zCNT structures with adsorption of one H atom (left) and one H_2_ molecule (right). The HER Gibbs free energy profile of (c) C_6_N_6_ and (d) C_12_N_4_. *U* = 0.73 V and *U* = 0.60 V are potentials provided by photo-generated electrons and applied on C_6_N_6_ and C_12_N_4_, respectively.

Driven by intrinsic electrostatic field in the core–shell structures, the protons produced in water splitting at the active site penetrate in to the CNTs^31^ and bond with the N atom of the inner CNNWs. As more protons enter the CNTs, HER takes place, producing H_2_ molecules in the CNTs, as shown in Fig. 4a and b.

Since HER occurred on CNNWs, we calculated HER performance on CNNWs to prove the feasibility of HER reaction on CNNW. Fig. 4c and d showed the HER performance of C_6_N_6_ and C_12_N_4_. Generally, the overall HER pathway is a two-electron process with one adsorbed intermediate H*. The activity of HER depends greatly on the Gibbs free-energy of this adsorbed intermediate, |Δ*G*_H*_|. The binding between H* and catalyst can't be too strong or too weak, in other words, the smaller the |Δ*G*_H*_| value, the higher the HER performance. Notably, the calculated Δ*G*_H*_ is −0.29 eV for C_6_N_6_ and −0.17 eV for C_12_N_4_, corresponding to low overpotentials (*η* = −0.29 V for C_6_N_6_ and *η* = −0.17 V for C_12_N_4_). Because the Pt crystal is the widely-used efficiently commercial HER electrocatalyst, we calculated the overpotential of the Pt(111) surface using the same method for comparison. As a result, the HER overpotential of the precious Pt catalysts with the value of −0.12 V is very close to that of the CNNWs. Notably, the |Δ*G*_H*_| values are lower than the potentials of the photo-generated energetic electrons in the CNNWs, 0.73(0.60) V and 0.51(0.34) V respectively for isolated C_6_N_6_ (C_12_N_4_) and C_6_N_6_ (C_12_N_4_) in core–shell structures, which are defined as the energy difference between the CBM and the hydrogen reduction potential. Therefore, under the potentials provided by photo-generated energetic electrons, for both C_6_N_6_ and C_12_N_4_, the two steps of HER are downhill in the free energy profiles, and thus can proceed spontaneously under light irradiation.

Finally, H_2_ molecules stored inside the CNTs by physically blocking the penetration of H_2_. CNTs have been widely studied for hydrogen storage, the store rates for the CNTs ranged from 0.01 wt% to 10 wt%, and CNTs would not have large deformations caused by hydrogen storages.^54^

Notably, the honeycomb network of carbon atoms (graphene and CNTs) allows the penetration of protons,^31,40^ and blocks O_2_/H_2_ and other functional groups. Therefore, the two half reaction of water splitting, HER and OER, take place inside and outside of CNTs, respectively. The produced H_2_ molecules are naturally separated from O_2_ and stably confined in CNTs. In this way, unnecessary reversible reaction in water splitting can be avoided and complete H_2_ reduction process can be realized.

## Conclusions

In summary, on the basis of density functional theory (DFT), we proposed one-dimensional core–shell CNNW/CNT structures to achieve efficient light absorption, photo-generated carrier separation, photocatalytic water splitting to generate hydrogen molecules and safe capsule hydrogen storage in a single system. It generates holes and electrons to be distributed respectively on active sites of the CNTs and the CNNWs by harvesting visible and ultraviolet light. Protons penetrate the wall of CNTs and HER takes place in the interior of CNT in the presence of CNNWs. The CNNWs exhibit excellent HER activity with the overpotentials of −0.29 V (for C_6_N_6_) and −0.17 V (for C_12_N_4_), which can be overcome by the potentials of the photo-generated energetic electrons in the CNNWs. The produced hydrogen molecules are naturally separated and stably stored inside the CNTs. These results are expected to pave a new feasible way for the production and storage of hydrogen molecules in CNTs.

## Conflicts of interest

There are no conflicts to declare.

## Supplementary Material

RA-012-D2RA02349K-s001
