# Supplementary material for: Photocatalytic hydrogen production and storage in carbon nanotubes: a first-principles study
Source: RSC Adv. 2022 Jun 8;12(27):17029–35. doi: 10.1039/d2ra02349k (PMC9175104; doi:10.1039/d2ra02349k)
Supplement: RA-012-D2RA02349K-s001 [file RA-012-D2RA02349K-s001.pdf]

Electronic Supplementary Material (ESI) for RSC Advances.
This journal is © The Royal Society of Chemistry 2022

Supporting Information for

Photocatalytic hydrogen production and storage in carbon nanotubes: A first-principle study

Xiaohan Song[a], Hongxia Bu[b], Yingcai Fan[c], Junru Wang[d], Mingwen Zhao[e*]

[a] *Shandong Institute of Advanced Technology, Jinan, Shandong, 250100, China*

[b] *College of Physics and Electronic Engineering, Qilu Normal University, Jinan, Shandong 250200, China*

[c] *School of Information and Electronic Engineering, Shandong Technology and Business University, Yantai, Shandong, 264005, China*

[d] *Department of Physics, Yantai University, Yantai, Shandong, 264005, China*

[e] *School of Physics and State Key Laboratory of Crystal Materials, Shandong University, Jinan, Shandong, 250100, China*

Corresponding author, E-mail: zmw@sdu.edu.cn

Text S1. The strategy of NAMD calculation

The nonadiabatic molecular dynamics (NAMD) simulations were carried out by the Hefei-NAMD code, where the real-time time-dependent KS equation framework was used to model the photo-excited carrier relaxation[1]. All structures were fully relaxed at 0 K, then we use the velocity rescaling to bring the temperature of the system to 100 K. After this, a 2.0 ps microcanonical ab initio molecular dynamics (MD) trajectory is then generated using a 1.0 fs time step. The NAMD results are based on an average over 100 different initial configurations obtained from the MD trajectory. For each chosen structure, we sample 2×10^4 trajectories for the last 1 ps.

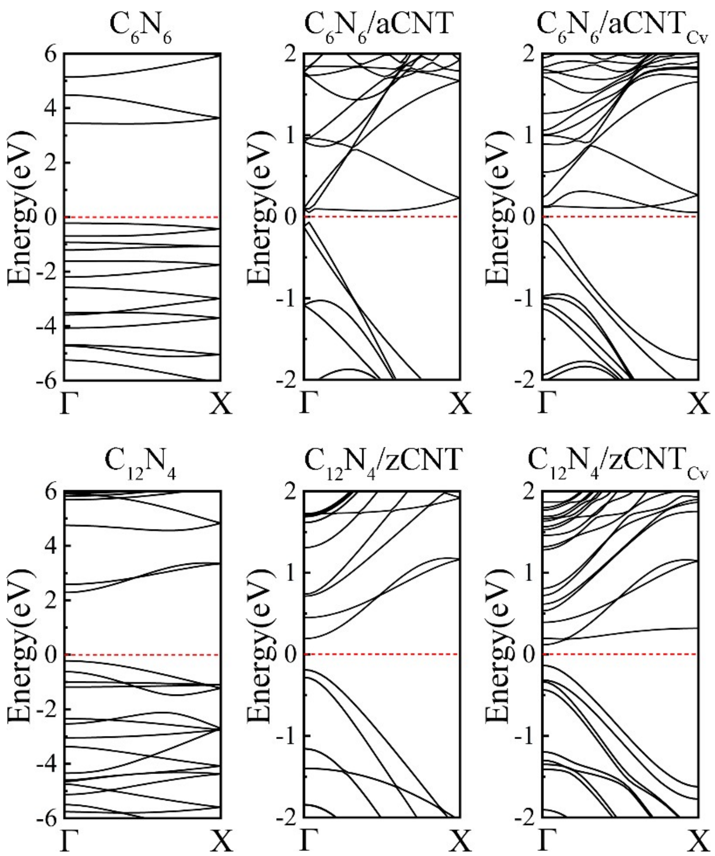

Fig. S1. Band structures of CNNWs (C_6N_6 and $C_{12}N_4$) and the core/shell structures (C_6N_6/aCNT, $C_{12}N_4$/zCNT, C_6N_6/aCNT$_{Cv}$ and $C_{12}N_4$/zCNT$_{Cv}$) calculated by using the HSE06 functional. The energy at the Fermi level was set to zero.

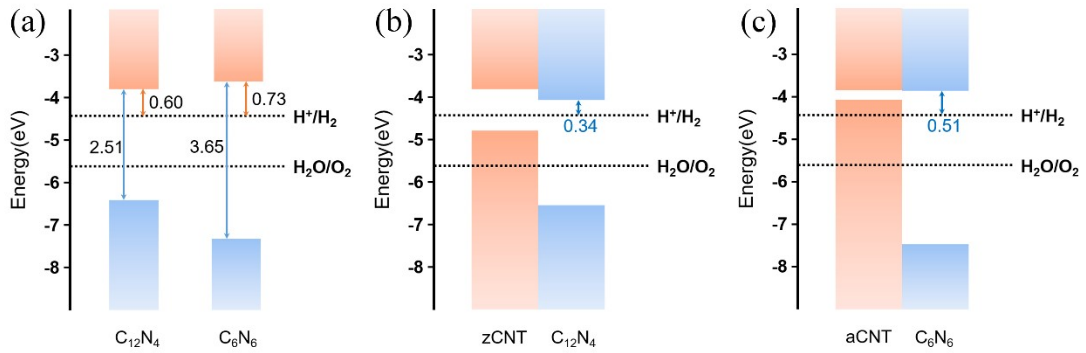

Fig. S2. Band edge alignment of (a) CNNWs (C_6N_6 and $C_{12}N_4$), (b) $C_{12}N_4$/zCNT and (c) C_6N_6/aCNT relative to the vacuum levels. The energy of the vacuum level was set to zero. The hydrogen reduction potential (H^+/H_2) and the water oxidation potential (H_2O/O_2) at pH 0 are -4.44 eV and -5.67 eV relative to the vacuum levels, respectively, which represented by the dotted lines.

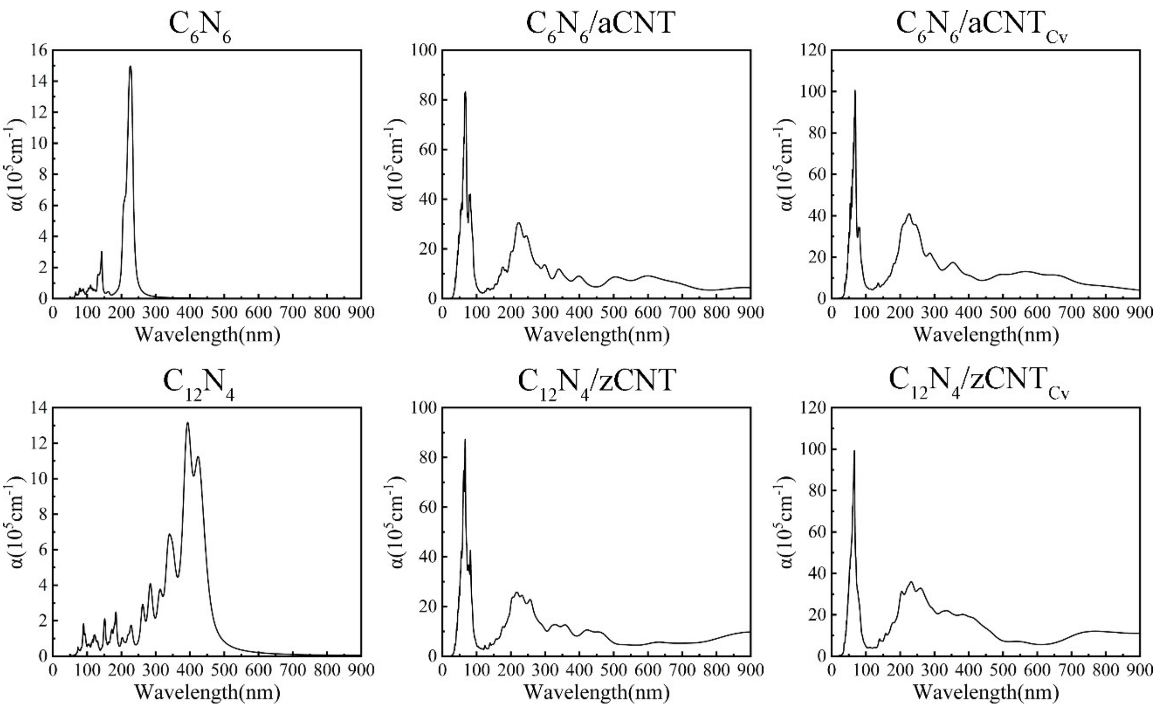

Fig. S3. Absorption coefficient of CNNWs (C_6N_6 and $C_{12}N_4$) and the core/shell structures (C_6N_6/aCNT, $C_{12}N_4$/zCNT, C_6N_6/aCNT$_{Cv}$ and $C_{12}N_4$/zCNT$_{Cv}$) calculated by using the HSE06 functional.

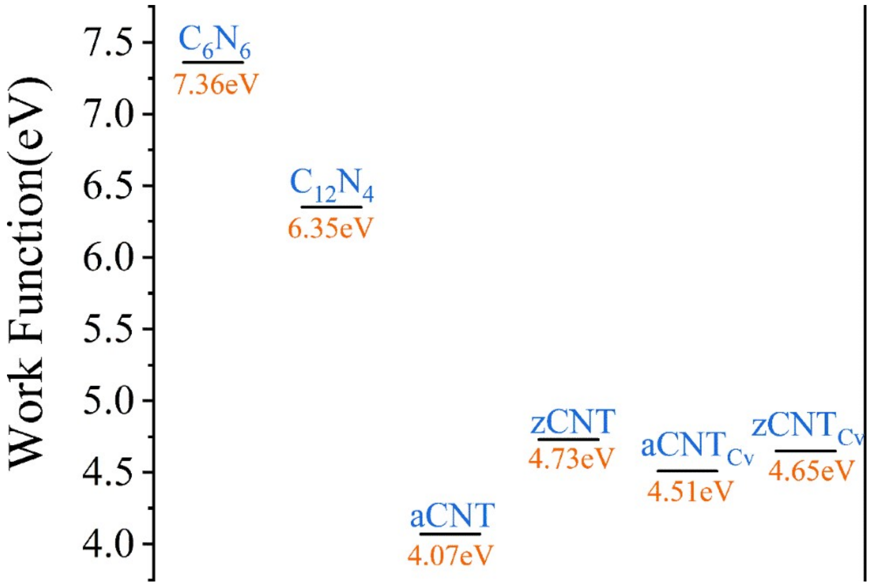

Fig. S4. The work functions of CNNWs (C_6N_6 and $C_{12}N_4$), CNTs (aCNT and zCNT) and CNTs with a vacancy ($aCNT_{Cv}$ and $zCNT_{Cv}$) calculated by using the HSE06 functional.

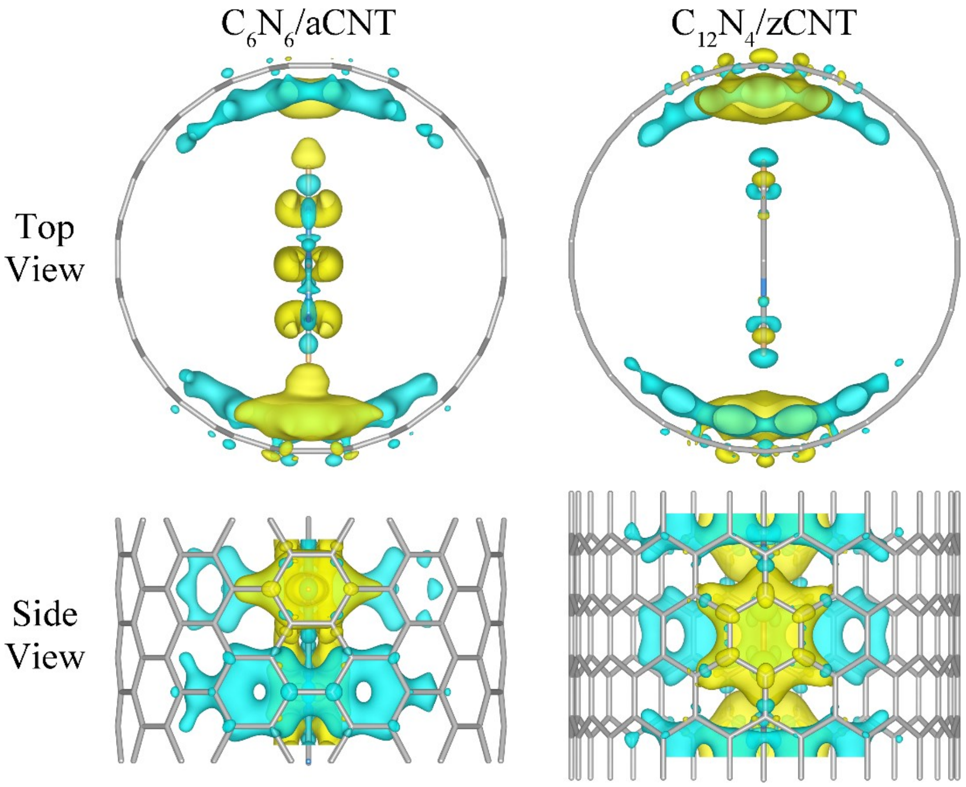

Fig. S5. Top and side views of charge density difference of the neutral core/shell structures (C_6N_6/aCNT and $C_{12}N_4$/zCNT). The isosurfaces are 4×10^{-4} e/Å^3. Yellow regions indicate electron accumulation, and blue regions indicate electron loss.

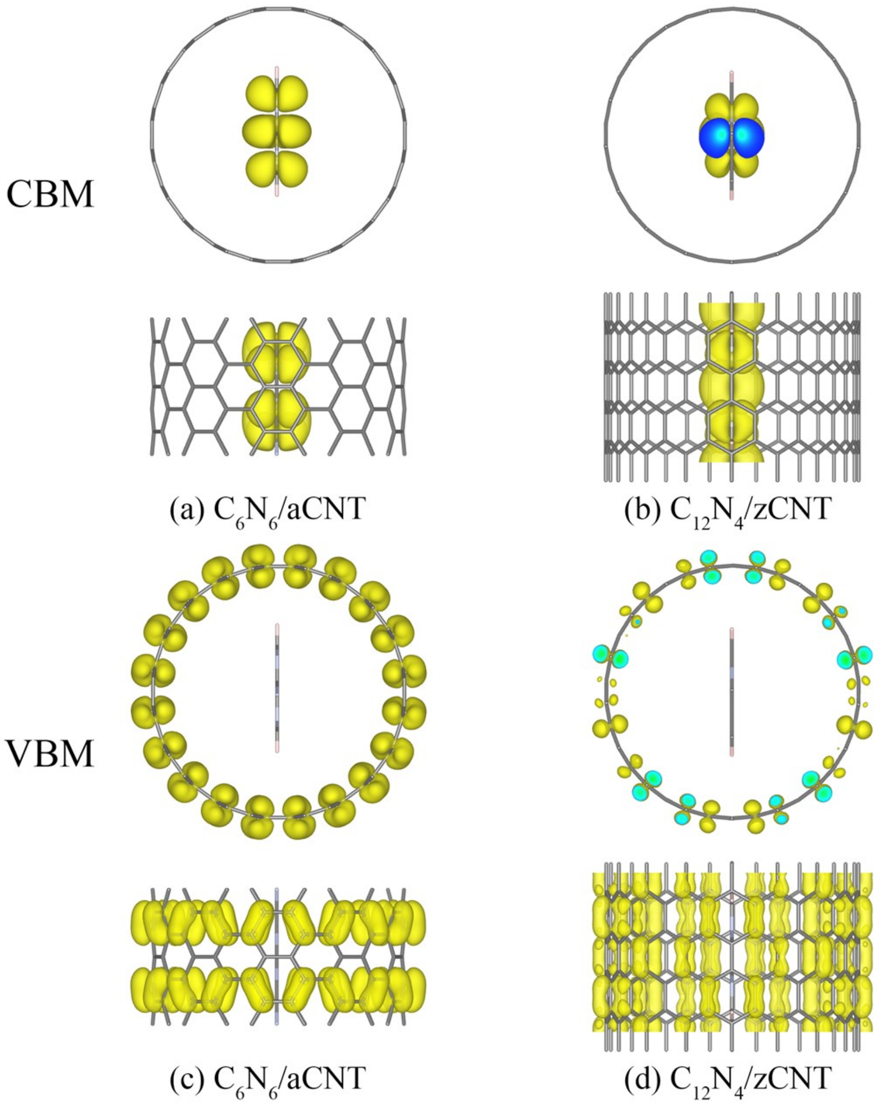

Fig. S6. Band-decomposed charge density distributions for the CBM and VBM of C_6N_6/aCNT and $C_{12}N_4$/zCNT structures. Yellow bubbles represent electron density with isosurface value of 3×10^{-3} e/Å^3 and 1×10^{-3} e/Å^3 respectively for C_6N_6/aCNT and $C_{12}N_4$/zCNT structures.

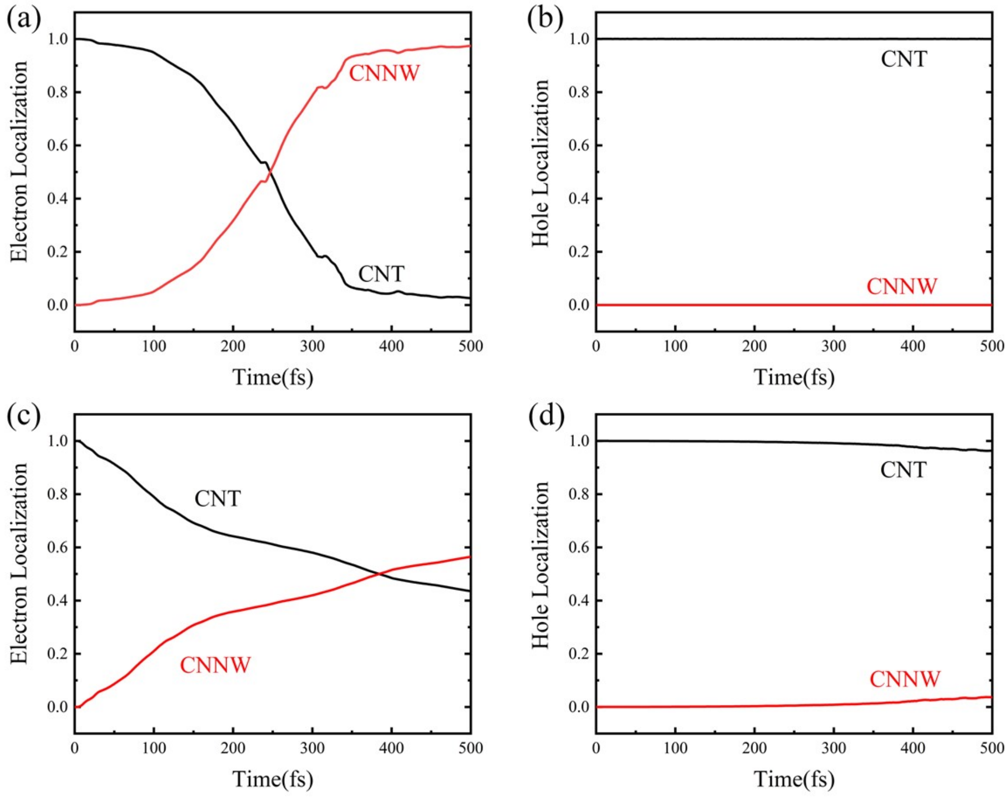

Fig. S7. The time dependent spatial charge localization of photogenerated (a) electron and (b) hole in the C_6N_6/aCNT structure at 100 K. The time dependent spatial charge localization of photogenerated (c) electron and (d) hole in the $C_{12}N_4$/zCNT structure at 100 K.

Table S1. Zero-pint energy correction (E_{ZPE}) of the adsorbates considered in this work.

E_{ZPE}(eV)	C_6N_6	$C_{12}N_4$
**H***	0.324	0.328

References

1. Q. Zheng, W. Chu, C. Zhao, L. Zhang, H. Guo, Y. Wang, X. Jiang and J. Zhao, *Wiley Interdiscip. Rev.: Comput. Mol. Sci.*, 2019, **9**, e1411.
